# Self-management of vaginal cube pessaries may be a game changer for pelvic organ prolapse treatment: a long-term follow-up study

**DOI:** 10.1007/s00192-022-05287-2

**Published:** 2022-07-16

**Authors:** Zoltan Nemeth, Szilard Kolumban, Roxana Schmidt, Peter Gubas, Kalman Kovacs, Balint Farkas

**Affiliations:** 1 Department of Gynaecology, Brothers of St. John of God Hospital Vienna, Vienna, Austria; 2 Department of Obstetrics and Gynaecology, University of Pecs School of Medicine, 17 Edesanyak Str., Pecs, Hungary; 3 Department of Obstetrics and Gynaecology, B-A-Z County Teaching Hospital, Miskolc, Hungary; 4 Member of the MTA-PTE Human Reproduction Scientific Research Group, Hungarian Academy of Sciences (MTA), Pecs, Hungary

**Keywords:** Vaginal pessary, Self-management, Long-term follow-up, Safety

## Abstract

**Introduction and hypothesis:**

Loss of anatomical support for the pelvic organs results in pelvic organ prolapse (POP). We hypothesized that daily self-management of a cube pessary might be a safe, feasible long-term treatment in women with symptomatic POP.

**Methods:**

A cohort of 214 symptomatic POP patients (stage 2+) were enrolled prospectively (January to December 2015). Each patient was size-fitted with a space-filling cube pessary and completed a questionnaire online or by phone ≥5 years after her initial fitting. Change in quality of life (QoL) was measured with the Patient Global Impression of Improvement (PGI-I).

**Results:**

Of 185 women included in our analyses, 174 (94%) were continuing to use their pessary 4 weeks post-insertion. Among those, 143 (82.2%) used the pessary successfully for ≥5 years. A large majority of these patients (88.8% [127 out of 143]) described their condition as much or very much improved compared with their pretreatment status (PGI–I). Adverse secondary effects (ASEs) were infrequent [15.4% (22 out of 143)]; when they did occur, they were mild, including smelly vaginal discharge (15 out of 22) and slight vaginal bleeding caused by the fitting procedure (6 out of 22).

**Conclusions:**

Daily self-management of cube pessaries was found to be a safe and effective treatment for improving POP-related symptoms and QoL in the long term.

## Introduction

Pelvic organ prolapse (POP) is a condition in which there is a loss of anatomical support for the pelvic organs that results in a deterioration in the quality of life (QoL) [1]. The exact incidence of POP is unknown and difficult to determine. The lifetime risk of women undergoing surgery for POP has been estimated to be within the range 11–19%, indicating that it is a relatively common condition [2, 3].

There are two major approaches to POP case management, namely, surgical treatment or conservative (nonsurgical) treatment, which may include modification of risk factors, pelvic floor exercises, hormonal replacement therapy, and pessary use. According to current guidelines, conservative treatment should be the first line of therapy [4–6]. Because most women can be fitted successfully with a pessary and their use has been associated with high satisfaction rates and only low rates of minor complications, it is appropriate to consider pessaries in all women presenting with bothersome POP and/or stress urinary incontinence [6]. Indeed, nearly two thirds of women with symptomatic POP choose to proceed initially with conservative case management [7].

The most common therapeutic tool used for POP internationally is a ring pessary, with a Gellhorn pessary being commonly recommended in more severe cases. Space-occupying pessaries, like the cube pessary, are generally considered to be more difficult for patients to manage than ring and sieve pessaries [8]. Notwithstanding, in a prior study in which we followed patients inserting and removing a cube pessary daily for a year, we found that it was safe and effective for most patients [9]. The advantages and disadvantages of daily self-management of a cube pessary are summarized in Table 1.

**Table 1:** Advantages and limitations of cube pessary use

Area	Advantage	Limitation
Effectiveness	Immediate	Not curative
Self-management	Patient has the ability to self-manage condition	Requires daily insertion and removal
Effects on/limitations in daily life	Elimination of symptoms enables participation in daily activities	Cannot be used during menstruation
Sexuality	Allows a sex life to be maintained	Needs to be removed before sexual activity
Accessibility	Easy to use, self-adjusted	Requires intact motor functions
Cost	Low price	–
Invasiveness	Non-invasive	–
Interaction with surgical treatment	Anti-POP surgery can be performed at any time	Can be unsuccessful after anti-POP surgery
Follow-up	Frequent follow-up not needed	–

The main aim of this study was to evaluate long-term (5-year) satisfaction and dropout rates in women using a cube pessary for symptomatic POP. Secondarily, we assessed the main reasons why patients discontinued cube pessary use.

## Materials and methods

### Study population

The target population were women who were started on a conservative treatment plan for POP at least 5 years prior to collection of data for this study, which occurred during May to August 2021. All recruited patients had their initial pessary fitting at one of two urogynecological outpatient clinics in Hungary (one in Győr and one in Budapest) between January and December of 2015. At their initial fittings, all of the participating patients were examined according to the guidelines established by the International Urogynecological Association. This prospective cohort study was approved by the University of Pecs Institutional Ethical Review Board (IV/7737-3/2021/EKU).

The inclusion criteria were symptomatic (bulge sensation in their vagina with or without symptoms of urinary, bowel, or sexual dysfunction) stage 2 or higher POP of the anterior, middle, and/or posterior compartments of the vagina, and successful fitting with a vaginal cube pessary (Dr Arabin®) for daily self-management. The exclusion criteria were any active infection of the pelvis or vagina (e.g., vaginitis, pelvic inflammatory disease), or physical or mental inability to manage independent use of the pessary, and discontinuation of pessary use for any reason within 4 weeks after fitting.

Participants completed a questionnaire online (link sent via e-mail) or by telephone interview if they did not have access to the internet. Of 214 patients who accessed the online questionnaire or were reached by phone, 185 completed the questionnaire. Of those 185 patients, 11 were excluded from this study because they did not have a successful initial fitting period leading them to discontinue pessary use within 1 month. Thus, data from the remaining 174 patients were included in our analyses.

### Data collection

The data were anonymized. For each patient, we recorded basic demographic data, including age and body mass index (BMI). Additionally, we recorded pertinent medical history data, including parity, method(s) of delivery, prior surgeries, size of the current cube pessary being used or reason for discontinuation and each patient's subjective self-assessment of POP-associated vaginal, bladder, bowel, and sexual symptoms. Pelvic anatomy alterations were classified according to the POP quantification (POP-Q) system [10] and all terminology used follow the recommendations of the International Continence Society.

The sizes of the fitted space-filling cube pessaries were designated 0–5, corresponding to the following respective diameters/volumes: size 0, 25 mm/15 cm^3^; size 1, 29 mm/24 cm^3^; size 2, 32 mm/30 cm^3^; size 3, 37 mm/42 cm^3^; size 4, 41 mm/60 cm^3^; and size 5, 45 mm/84 cm^3^ [9]. At the fitting stage, we emphasized that pessary therapy is like wearing eyeglasses in that they are medically assistive devices that can eliminate symptoms immediately during their use. We inform patients that a pessary is easy to use and cost effective, and that daily pessary use should not disturb sexual activity [9]. Appropriate pessary size was determined for each individual with the goal of the pessary being large enough to resolve POP symptoms while being small enough to avoid discomfort [11]. The patients received detailed instructions on pessary use and care, with particular attention being given to the removal technique. Postmenopausal women were recommended to apply local estrogen (estriol, Ovestin®) replacement therapy twice a week. Patients were told to return for a control check-up at any time, if they have a complaint, but no later than once a year.

Changes in QoL after pessary use were assessed with the validated Patients Global Impression of Improvement (PGI-I) scale [12]. The patients were asked whether they had experienced any adverse secondary effects (ASEs) of pessary usage. The treatment plan was considered successful for 5 years if the patient used the pessary regularly and wished to continue its use. Patients were considered lost to follow-up if they could not be contacted either online or by telephone.

### Statistical analysis

Statistical analyses were performed in SPSS version 20 (IBM, Armonk, NY, USA) at the University of Pecs Institute of Bioanalysis. Continuous measurement variable data are presented as means with standard deviations (SDs), whereas categorical data are presented as number of observations or percentage values. Fisher’s exact test was applied to independence analyses performed between categorical variables. Statistical significance was set at *p* < 0.05 or *p* < 0.1, as indicated.

## Results

### Demographics

The questionnaire response rate was 86.45% (185 out of 214 patients who agreed to complete a questionnaire). Of the 185 responders, 11 had an unsuccessful initial fitting period (<1 month after initiation), leaving 174 study participants in the final sample (94% of the responders).

The mean (±SD) age of the study sample was 61 ± 13 years (range, 33–91), with a mean BMI of 25.99 kg/m^2^ ± 4.06 (range, 15.63–43.28). Obstetrical history review revealed a mean birth rate of 1.5 births per woman (range, 0–4), with a cesarean section rate of 0.1% (18 out of 174) and an operative vaginal delivery rate of 0.09% (vacuum extraction, 12 out of 174; forceps delivery, 3 out of 174); both of these birth intervention rates were significantly lower than the national average (*p* < 0.01). Two thirds of the participants were postmenopausal (66.66%; 116 out of 174) at the time of the fitting. Gynecological anamnesis showed a 12.1% hysterectomy rate (21 out of 174), of which 57.1% were abdominal operations (12 out of 21) and 42.9% were vaginal operations (9 out of 21). Almost a third of the participants (32.2%; 56 out of 174) underwent anterior and/or posterior repair and only 4 patients underwent another kind of pelvic floor surgery (1 transvaginal mesh, 1 Manchester repair, and 2 ventrofixation). The characteristics of premenopausal and postmenopausal patients are compared in Table 2. The groups were similar with the exceptions of the postmenopausal group being older and having a higher mean BMI.

**Table 2:** Comparison of characteristics across pre- (age <55 years) and postmenopausal (age ≥ 55 years) patient groups

Characteristic	Pre-menopausal (*N* = 58)	Post-menopausal (*N* = 116)	*p*
Mean age ± SD, years	47 ± 5.71	68 ± 8.0	<0.001
Mean BMI ± SD, kg/m^2^	24.0 ± 4.56	27.0 ± 4.0	<0.001
Parity, median (min; max)	2 (0;4)	2 (0;5)	0.868
Obstetrical data			
Caesarian section, %	18.96	6.03	0.031
Vacuum extraction, %	10.34	5.17	0.205
Forceps delivery, %	1.72	1.72	>0.999
Gynecological data			
Abdominal Hx, %	0	11.2	0.023
Vaginal Hx, %	1.72	6.89	0.122
Colporrhaphy rate, %	20.30	37.06	0.138
Other anti-POP procedure, %	0	2.40	0.554

*BMI* body mass index, *Hx* hysterectomy, *POP* pelvic organ prolapse, *SD* standard deviation

### Cube pessary usage

More than four fifths of the study participants (81.2%; 143 out of 174) reported using the cube pessary successfully for at least 5 years after the initial fitting. Most of these women (83.2%, 119 out of 143) used it daily with a high satisfaction rates, whereas a small number of these women used it a few days each week (8.4%) or only occasionally (8.4%). All but one of the participants described the self-fitting procedure as easy or very easy, indicated that the intravaginal presence of the pessary was not noticeable, and reported that use of the pessary eliminated their POP symptoms.

The 5-year discontinuation rate was 17.8% (31 out of 174), with discontinuation being more than twice as common among premenopausal women (29.3% [17 out of 58]) than among postmenopausal women (12.1% [14 out of 116]; *p* < 0.05). Of the 58 premenopausal women who discontinued pessary use during the observation period, 5 (8.6%) indicated that they did so because they became symptom free owing to a combination of therapies, described in detail previously [13]. Patients' reasons for discontinuation and the frequencies of each reason are summarized by age group and time of usage in Table 3.

**Table 3:** Reasons reported by patients for discontinuing cube pessary use

Time used	*n*	Reason	*n*	Anti-POP surgery (*n*)
<1 year	15	Became symptomless	3	1
		Chose another treatment^a^	2	
		Discomfort	9	
		Urinary incontinence	1	
>1 year	16	Became symptomless	2	6
		Chose another treatment^a^	6	
		Urinary incontinence	1	
		Discomfort	7	

^a^Other treatments included other conservative treatment options, namely laser therapy, electrostimulation therapy, and/or pelvic floor muscle exercises

**Fig. 1:**
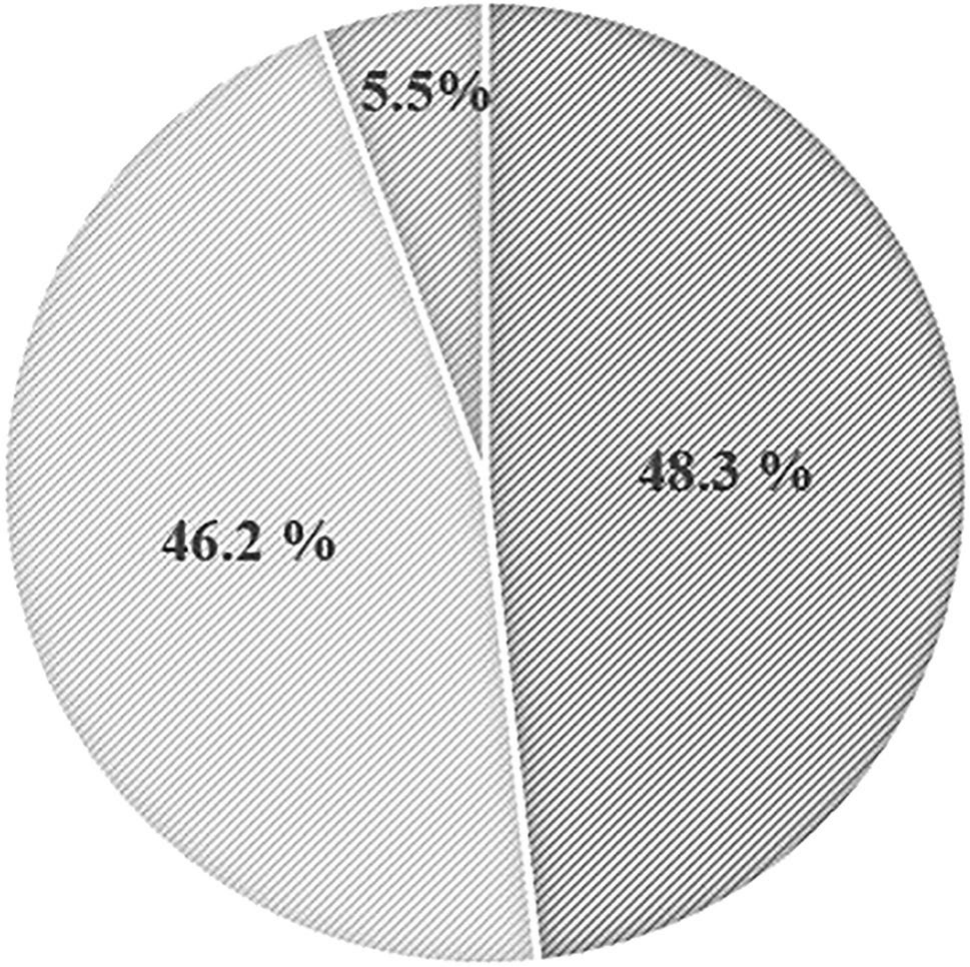
Cube pessary adjustments in women with symptomatic pelvic organ prolapse during the 5-year observation period of this study. The percentages of participants who had their pessary size reduced, increased, and never changed are shown in *dark gray*, *medium gray*, and *light gray* pie sections respectively. Note that the size was often decreased but rarely increased

The average cube pessary size was initially (37 mm/42 cm^3^) but was reduced to 2 (32 mm/30 cm^3^) by the end of the 5-year observation period, consistent with POP improvement and an associated decrease in excess vaginal space owing to other conservative treatments. Indeed, nearly half of the patients who continued using their cube pessaries throughout the observation period (69 out of 143) reported having a pessary size reduction during the 5-year observation period, whereas most of the remaining patients (66 out of 143) retained the same pessary size throughout and relatively few patients (8 out of 143) transitioned to a larger size pessary (Fig. 1).

### Symptoms and QoL

Patients who were actively using the cube pessary throughout the observation period reported high satisfaction rates 5 years after the initial fitting. Self-reported symptom evolution data are reported in Table 4. Notably, the overwhelming majority of patients (88.8% [127 out of 143]) reported experiencing substantial improvement (very much better or much better) in their condition, whereas no patients reported experiencing substantial worsening of their condition during the 5-year study period. Only a single patient reported no change and one other patient reported slight worsening.

**Table 4:** Self-reported pelvic organ prolapse symptom scores among active cube pessary users after 5 years of use (*N* = 143)

Questionnaire response (rating)	Symptom, %, *n*					
	Bulge seen or felt		Bladder symptoms		Bowel symptoms	
Very much better (1)	71.3	102	32.2	46	9.8	14
Much better (2)	23.1	33	45.5	65	19.6	28
A little better (3)	0.0	–	0.0	–	0.0	–
No change (4)	2.8	4	3.5	5	8.4	13
A little worse (5)	0.0	0	0.7	1	0.7	1
Much worse (6)	0.0	–	0.0	–	0.0	–
Very much worse (7)	0.0	–	0.0	–	0.0	–
Did not have this symptom before	2.8	4	18.2	26	60.8	87

### Adverse secondary effects

During the observation period, 15.4% of the participants (22 out of 143) reported experiencing ASEs. The most common ASE among those who reported ASEs was mild vaginal discharge (68.1% [15/22]), followed by slight vaginal bleeding due to vaginal epithelial lesions caused by the fitting process (27.3% [6 out of 22]). One patient reported experiencing lower abdominal pain during usage. No severe complications were reported.

## Discussion

In the current prospective study, we report for the first time to our knowledge, the long-term experience of symptomatic POP patients with self-management of cube pessaries. The vast majority of the participants used the pessary successfully for at least 5 years and nearly nine tenths of those who did so describe their condition as being much or very much better after 5 years of pessary use. The high continuation rate was a surprising result. There might be three potential explanations for that:

1. The application of vaginal cube pessary immediately overcomes the POP-related symptoms, and after proper fitting, the vast majority of users can forget their problem during the day.
2. Regular users describe self-management of vaginal cube pessaries as being either very easy or easy.
3. Owing to the nightly removal of the cube pessary it provides opportunity for the vaginal epithelium to regenerate; therefore, the incidence of side effects is found to be low, and if they do appear, the treatment requires minimal effort and can be resolved after a couple of days’ rest.

Although pessaries are a widely accepted conservative treatment modality for symptomatic POP, there are limited data available on their self-management and the long-term outcomes after their use. Previously, we presented the first 1-year outcomes of cube pessary self-management [9]. Kearney at al. developed a program designed to teach women how to self-manage their pessaries with the aim of improving patient experience and reducing outpatient attendances. They found that, after 6 months, women practicing self-management reported higher levels of convenience, accessibility, support, and comfort than those who had their pessaries changed by a health care professional [14]. In a study examining 289 women fitted with ring pessaries for symptomatic POP, Manonai et al. found that only 61.7% were still using them 5 years later and that self-care was the only significant factor that was predictive of continued compliance for 3 years [15]. In a study of 93 patients who opted for self-management of Gellhorn pessaries to treat symptomatic POP, Chien et al. found that only 47.2% of the study participants were still using their pessaries after 5 years [16]. Our relatively high continuation rate in the present study (82.2%) compared with those reported previously could be attributed, at least in part, to the use of a different type of pessary.

Common reasons for discontinuation of doctor-led long-term pessary treatment include heavy discharge, bleeding, experiencing discomfort during pessary changes, and disruption of sexual activity. In a study of 273 women fitted with a ring pessary, Sarma et al. found that 56% of the patients experienced complications and that only 14% were still using their pessaries after 6 years [17]. In our study cohort, only 15.2% of participants reported ASEs, all of which were minor. Removal of the pessary before going to sleep every day may allow vaginal tissues to heal and regenerate nightly and thus prevent the development of serious complications.

In a 5-year study of 151 patients being treated mainly with ring pessaries on a doctor-led basis, Lone et al. report a high success rate (86.1%) similar to that observed in the present study. They found that most failures (73.8%) occurred within 4 weeks of pessary insertion. Overall, 12.1% of the women in Lone et al.'s study experienced minor complications, similar to the ASE rate observed in the present study. The authors posited that the relatively old age of their patient sample (median age, 70 years) could be the reason for their high success rate [18]. In our study, the median age was 61 years and only 6% (11 out of 185) of the participants discontinued pessary use within 4 weeks of their initial fitting. In a study of 265 patients fitted initially with ring pessaries, which were replaced with Gellhorn pessaries if the ring pessaries were failing, Ma et al. [19] observed a 5-year success rate of 75.3%. They identified a lack of self-management ability as an important discontinuation risk factor [19]. We agree with this suggestion and believe that pessary therapy success rates may be improved in the future with pessaries amenable to self-management.

Commonly, pessary use is considered a suitable treatment primarily for elderly women who are not eligible candidates for surgery or who do not wish to undergo surgery and for women who may yet bear children [17, 20]. In our current study, almost one third of the long-term cube pessary users were premenopausal (age ≤ 55 years), consistent with previously reported data [21]. Our findings support the recommendation that self-managed vaginal pessaries should be among the treatment options primarily offered to premenopausal women with symptomatic POP. In our experience, the success of pessary self-management depends largely on patient willingness to adhere to the treatment plan and a good pessary-size fit.

Pessary therapy is often viewed as a means of delaying surgery. However, older age is associated with greater risks of anesthesia-related ASEs and postoperative recovery complications. Thus far, however, there are no available data addressing the question of whether delayed surgery for POP increases the risk of bad outcomes or complications.

In a previous study [22], we found that 21.5% of women who attempted a pessary fitting for POP recurrence after vaginal/pelvic floor reconstructive surgery could not be fitted successfully owing to surgically altered anatomy. The probability of failure was 18.8% after one surgery and 27.3% after two surgeries. Thus, it is important for gynecologists to be aware that surgery remains an option after first-line pessary therapy if it fails, whereas first-line surgery will preclude successful subsequent pessary use in a substantial minority of patients.

In a prior study we found that excess vaginal space is a marker of prolapse stage and a risk factor for POP recurrence [23]. Our finding of common cube-pessary downsizing suggests that the intervention might actually improve POP in addition to relieving symptoms. That is, excess vaginal space, which correlates with the severity of an expanded genital hiatus, may become reduced with long-term cube-pessary use.

The cube pessary is a support pessary that maintains its position by creating a vacuum effect on its six concave surfaces within the vagina. Although some have speculated that the constant suction might lead to erosion and fistulation of the vaginal walls [24], we have not observed a single case of genital fistula formation in cube pessary users in our practice, and mild vaginal erosion occurred in only 4.19% of the participants in this study (6 out of 143). To date, no major complications of cube pessary use have been reported in patients who remove it daily. There is a single case reported wherein the user developed a recto-vaginal fistula after failing to remove a cube pessary for 10 weeks [25].

To the best of our knowledge, this the first long-term follow-up study of self-managed cube pessary use in the literature with a substantial sample size. Data on all major aspects of treatment are reported herein, including POP severity improvement, POP-related symptom improvement, ASE occurrence, and QoL.

A potential bias can be the specific type of cube pessary used by our study group, as the commercially available cube pessaries produced by different companies vary in both quality (including shape, design) and raw material (from rigid plastic to tissue-friendly silicone), which in our opinion can significantly influence the success of therapy, and patient satisfaction. An objective comparison of different vendors’ products is required in a future study.

This study had two notable limitations. First, we did not use a translated and validated questionnaire to assess symptom evolution (e.g. the 20-item Pelvic Floor Disability Index) because none is yet available in the Hungarian language. Second, we did not have the chance to examine the patients directly at the conclusion of the observation period owing to COVID-19 pandemic restrictions.

## Conclusion

Daily self-managed cube pessary use was found to be safe and effective. This treatment modality offers a convenient and cost-effective method of treating POP-related symptoms and thus improving QoL in the long term. Given our clinical experience and the present empirical data, it is our view that self-management is a critical factor in the long-term success of pessary treatment.
